# Safety use of high frequency oscillatory ventilation in transport of newborn infants affected by severe respiratory failure: preliminary data in central Tuscany

**DOI:** 10.1186/s12887-022-03393-0

**Published:** 2022-06-10

**Authors:** Gilda Belli, Ilaria Dovadola, Elettra Berti, Letizia Padrini, Elisabetta Agostini, Lisa Serafini, Anna Ingargiola, Gabriella Gabbrielli, Elena Sandini, Angelo Azzarà, Serena Catarzi, Maria Luce Cioni, Letizia Petrucci, Filomena Paternoster, Marco Moroni

**Affiliations:** 1 Neonatal Intensive Care Unit, San Giovanni di Dio Hospital, Florence, Italy; 2 Department of Women’s and Children’s Health, University of Padova, Padua, Italy; 3 Neonatal Intensive Care Unit, Neonatal Emergency Transport Service, A. Meyer University Children’s Hospital, Florence, Italy

**Keywords:** Neonatal transport, High frequency oscillatory ventilation, Newborn, Respiratory Failure

## Abstract

**Background:**

Neonatal Emergency Transport Services play a fundamental role in neonatal care. Stabilization before transport of newborns suffering from severe respiratory failure is often a challenging problem and some critically ill infants may benefit from High Frequency Oscillatory Ventilation (HFOV) as rescue treatment. In these cases, transition to conventional ventilation for transport may cause a deterioration in clinical conditions. HFOV during neonatal transport has been only exceptionally used, due to technical difficulties. Since May 2018, a new neonatal transport unit is available at the Neonatal Protected Transport Service of the Meyer University Hospital in Florence, equipped with a pulmonary ventilator capable of delivering HFOV. Therefore, we conducted an analysis on patients transferred in HFOV to Neonatal Intensive Care Unit (NICU), in order to evaluate the safety and feasibility of its use during neonatal transport.

**Methods:**

A retrospective analysis was performed reviewing medical records of the neonates transported by Meyer Children Hospital’s Neonatal Transport Service between May 2018 and December 2020, and newborns treated with HFOV during ground neonatal transport were identified. Safety was assessed by the comparison of vital signs, hemogas-analysis values and pulmonary ventilator parameters, at the time of departure and upon arrival in NICU. The dose of inotropes, the main respiratory complications (air leak, dislocation or obstruction of the endotracheal tube, loss of chest vibrations) and the number of deaths and transfer failures were recorded.

**Results:**

Out of the approximate 400 newborns transported during the analysis period, 9 were transported in HFOV. We did not find any statistically significant difference in vital parameters, hemogas-analytical values and pulmonary ventilator settings recorded before and after neonatal transport of the nine patients’ parameters (*p* > 0,05). No patient required additional inotropes during transport. No transport-related deaths or significant complications occurred during transport.

**Conclusions:**

The interest of our report is in the possibility of using HFOV during inter-hospital neonatal transfer. As far as our experience has shown, HFOV appears to be safe for the transportation of newborns with severe respiratory failure. Nevertheless, further larger, prospective and multicentre studies are needed to better evaluate the safety and efficacy of HFOV during neonatal transport.

## Background

Neonatal Emergency Transport Services (NETSs) play a fundamental role in reducing the risks of inconveniences during transport of infants which requires transfer to centres equipped with Neonatal Intensive Care Unit (NICU) [1–3], either because of foetal malformations or pathologies, whenever it is not possible to transfer pregnant women before childbirth, or in case of disease which result after childbirth. Assisted neonatal transport is also useful for infants already in NICU, who need to be transferred to other centres for the execution of diagnostic or therapeutic procedures not available on site.

Stabilization before transport of infants suffering from severe respiratory failure is often a challenging problem for the NETS team [4, 5]: in some cases, these patients are poorly responsive to Conventional Mechanical Ventilation (CMV) or ventilator parameters harmful to lung dynamics are necessary. These patients could benefit from transition to high frequency oscillatory ventilation (HFOV), since it is considered a lung protection strategy [6]. Some studies have been carried out on the use of HFOV as an elective mode of ventilation in neonatal respiratory pathology, obtaining good results both in terms of ventilator and hemodynamic parameters [7–10], but evidence of its effectiveness as a first choice ventilatory method is still poor [11]. HFOV, however, finds greater success as a rescue therapy in infants with severe respiratory distress, not responsive to CMV, due to different pulmonary diseases [7, 8, 12–19].

Nevertheless, the use of HFOV during neonatal transport has been limited by important technical problems: the system must be independent and equipped with an internal battery and medical gas cylinders able to provide sufficient service autonomy, in order to allow the newborn infant to be stabilized at the birth point and then transferred. Hence, the rare reports in literature concerning the use of High Frequency Ventilation (HFV) in neonatal transport refer mainly to sporadic experiences and different modalities, such as High Frequency Jet Ventilation (HFJV) and High Frequency Percussive Ventilation (HFPV) [20–22]. As far as we know, a single study has investigated HFOV in neonatal transport [23] but a limited autonomy of the HFOV device prevented a wide application in neonatal transport. The intrinsic characteristics of the high oscillatory frequency ventilator used so far have presented additional limits when used in neonatal transport: large dimensions, considerable weight, and electromagnetic interference, often incompatible with the neonatal transport environment [21]. Indeed, Panico and collaborators, in 2015, in an essay on new technologies applied to neonatal transport stated that “transport with HFOV is not an option at this time possible, unless it has an external charging system” [24].

Since May 2018, a new neonatal transport unit is available at the Neonatal Protected Transport Service of the Meyer University Hospital in Florence, designed to overcome these problems: it is equipped with a pulmonary ventilator also capable of delivering HFOV. The aim of the present study is to evaluate the safety and feasibility of HFOV use during neonatal transport.

## Methods

A retrospective study was conducted, analysing the medical and transport records of newborns transferred by the NETS of Central Area of the Tuscany Region, between May 2018 and December 2020. The service provided ground neonatal transport, covers a total of 9 related centres and in the catchment area there were 12,819 births in 2018: in that year, emergency neonatal transport was carried out for a total of 225 patients. In the study period (from May 2018 to December 2020) a total of 462 neonatal transportations were necessary in the Central Area of the Tuscany Region.

Infants treated with HFOV during transport were selected and recruited into the study. In all the patients, HFOV was chosen either because the patient had already been stabilized by means of HFOV at the place of birth or because of conventional ventilation failure, intended as Sat02 persistently lower than 90% despite high ventilator parameters like Peak Inspiratory Pressure > 25–28 mmHg, depending on the clinical features of each newborn (kind of respiratory disease, gestational age, birth weight, etc.), Fi02 100%, respiratory frequency (FR) > 50 apm. In these cases the severity of the respiratory pathology did not allow the transfer to the NICU using other ventilator strategies.

### Characteristics of the transport unit

**Fig. 1:**
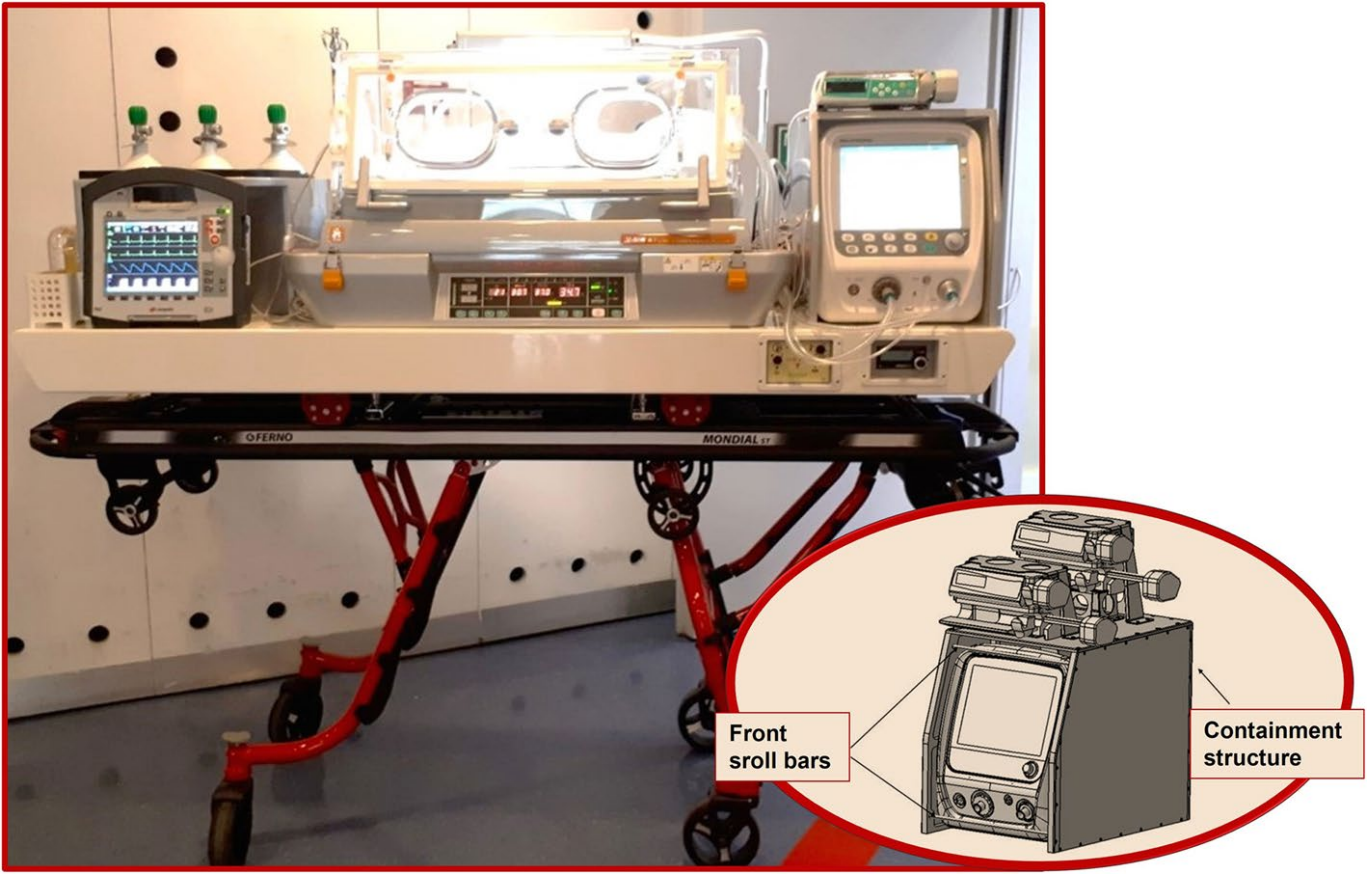
Image of the whole transport module and schematic draft of the structure to accommodate pulmonary ventilator on transport platform

The Neonatal Transport Unit used is a fully certified system for ground transport and integrates all the functions and devices necessary for safe and protected transport of critical infants. The innovation of the structure consists in the realisation of a single platform on which they find accommodation and connect all the necessary equipment for neonatal intensive care, including three compartments housing the medical compressed air and oxygen cylinders with a capacity of 3 L, and one of 2 L of nitrogen monoxide. The system also allows to be supplied through external sockets of medical gases in the ambulance or at the birthplace through special connection tubes: in this case, the gas supply from the cylinders is automatically excluded. On the right side of the platform, there is the innovative structure built to accommodate a pulmonary ventilator, showed in Fig. 1: it is an aluminum frame designed to make the respirator rest, mounted on four rubber feet, contained by the frame on the back side and on the right and left sides.

On the front side, where the controls and settings are present, two transverse bars have been arranged, fixed with bolts, which block the device, but do not prevent the display of the patient's parameters. On the back side of the structure, there are tubes for air/oxygen supply and power supply from the platform.

The pulmonary ventilator used in this study is an Acutronic Fabian HFOi: it is a continuous flow, limited in pressure, device, with volume control, controlled by microprocessors, which combines the most advanced functions for respiratory support of infants. It can provide both invasive and non-invasive CMV and HFOV. The latter is achieved through a large, high-power active speaker membrane, easily controlled by the operator: the system for HFOV is fully integrated into the inspiratory port and no additional connectors are required. The ventilator is equipped with its own built-in batteries and the device continuously calculates the remaining charging time and displays it on the information bar: the autonomy declared by the manufacturer is about 2.5 h, when used in conventional ventilation mode, and about 1 h, if used in HFOV. Nevertheless, if prolonged use at the birth point is expected, it is sufficient to connect the Neonatal Transport Unit to a power supply; moreover, during transfer by ambulance, the unit is always attached to the power grid of the vehicle. The ventilator batteries fully recharge in a maximum time of 5 h. When the device works with internal battery, the amplitude of the HFOV is limited to 60 mbar. As far as the medical gas reserve is concerned, it is difficult to calculate their consumption, because there are many variables involved, mainly the flow set and the FiO_2_ delivered. In the extreme case of exclusive use of only one type of gas (Inhaled Oxygen Fraction 21% or 100%) with inspiratory and expiratory flows equal to 8 L/minute, the minimum life of a cylinder can be calculated as being of/at about 75 min, according to the following formula:

- air or oxygen cylinder capacity: 3 L
- gas volume in the cylinder: 600 L (200 bar × 3 L)
- ventilator flow: 8 L/min.
  - ➔ = 75 min.

However, during the infant’s stabilization at birthplace, it is also possible to connect the system to the hospital's gas network, through the appropriate connection tubes; finally, spare cylinders are available in the ambulance, for replacement in case of exhaustion. During the journey, the ventilator is always connected to the gas network of the ambulance. The platform also includes an easy-to-use inhaled nitrogen monoxide (iNO) flow regulator, using digital mass flow technology.

### Analysis methods

The medical records of patients, who complied with the inclusion criteria, were revised and sex, gestational age, birth weight, Apgar index at the first and fifth minute of life, respiratory pathology were collected. Transport data was also documented: transport time, weight, and age of the newborn at the time of Neonatal Protected Transport (NPT). Transport time is the interval, expressed in minutes, which the transport team took from departure from the hospital of birth up to arrival at our tertiary level NICU.

To assess the safety of HFOV during the transfer of infants, the following variables were considered:

- vital parameters: heart rate (HR), expressed in beats per minute (bpm), measured with ECG monitoring; mean blood pressure (MBP), expressed in mmHg, measured bloodlessly by the multiparametric monitor or calculated according to the formula MBP = (2(DBP) + SBP)/3, where DBP is the mean diastolic pressure and SPB the mean systolic pressure; oxygen saturation rate (SaO2) of haemoglobin, detected at the upper right limb.
- capillary or arterial Hemogas-Analysis (EGA) parameters: pH; partial carbon dioxide pressure (PaCO_2_), expressed in mmHg; excess bases (EB), in mmol/l; bicarbonates (HCO3-), in mmol/l. Among the hemogas-alytic parameters detected we did not include partial oxygen pressure (PaO_2_) for lack of uniformity between the sources of sampling (arterial or capillary), before and after transport. As a parameter for oxygenation, SatO2 was considered.
- pulmonary ventilator parameters: mean airway pressure (P_aw_), expressed in cmH_2_O; inspiratory fraction of oxygen (FiO_2_), expressed as a percentage; Frequency (Fr), expressed in Hz; Amplitude or Power (ΔP), expressed in mbar; possible administration of inhaled nitrogen monoxide (iNO), in parts per million (ppm).

All variables were recorded at the time of departure (labelled as “pre-NPT”) and upon arrival (labelled as “post-NPT”) in NICU. The pre-NPT parameter is the last available value recorded on neonatal transport file before departure, and the post-NPT parameter is the first available value after arrival.

The comparison of the parameters was performed with two-tailed Student t-test for paired data. The values were expressed as an average value ± standard deviation (DS).

Doses of inotropic drugs (Dopamine, Dobutamine, Adrenaline, Norepinephrine) and bicarbonates administered have been recorded. In addition, any complications that arose during transport were detected, such as air leakage syndromes, dislocation or obstruction of the endotracheal tube and the loss of chest vibrations, visually observable. Finally, the number of deaths and transfer failures, considered as interruption of transport and return to the transfer centre, for the development of hemodynamic instability of the patient or exhaustion of ventilator autonomy, was also noted.

## Results

From May 1, 2018, 9 infants with severe respiratory failure were transferred using HFOV from birthplace to the NICU of Meyer University Children's Hospital, in Florence, and were enrolled in our study. They showed a mean gestational age of 36 weeks ± 3.5 days, and a mean birth weight of 2772 ± 532 g. All patients presented serious respiratory failure, with variable aetiology, as reported in Table 1.

**Table 1:** Characteristics of the patients enrolled

Patient n°	Sex	Gestational Age (weeks + days)	Birth weight (g)	Apgar Index		Respiratory Disorder
				1^st^ minute	5^th^ minute	
1	Female	27 + 5	1950	2	4	CCAM Rt
2	Male	36 + 5	3010	5	5	CDH Rt
3	Male	40 + 5	3000	5	8	CDH Lt
4	Male	38 + 2	2620	8	9	CDH Rt
5	Male	33 + 4	2310	2	7	CDH Lt
6	Female	38 + 0	3160	2	4	PA-PTX
7	Female	38 + 0	3260	8	10	PTX Rt
8	Male	39 + 4	3476	6	7	MAS
9	Male	32 + 6	2170	3	2	PA

*Abbreviations: CCAM* Congenital Cystic Adenomatoid Malformation of the lung, *CDH* Congenital Diaphragmatic Hernia, *PA* Perinatal Asphyxia, *PTX* Pneumothorax, *MAS* Meconium Aspiration Syndrome, *Right* Rt, *Left* Lt

The characteristics of patients have been detailed in Table 1.

The mean patient transport time was 21.11 ± 10.2 min. The maximum transport time resulted 45 min. With regards to age at time of neonatal transfer, infants were transported between a minimum of 3 and a maximum of 34 h of life. The average weight at the time of transport was 2767.78 g.

Of the 9 infants transferred using HFOV, 6 (patients numbered 1 to 6) were already treated with HFOV at the hospital of birth. For these patients, conversion to CMV, for the transport phase would have resulted likely in hemodynamic and respiratory instability, thus making them difficult to transport to NICU. The remaining 3 infants (patients 7 to 9) were placed in HFOV by the NETS team, as rescue treatment, given the severity of the clinical picture and the failure of conventional ventilation.

**Fig. 2:**
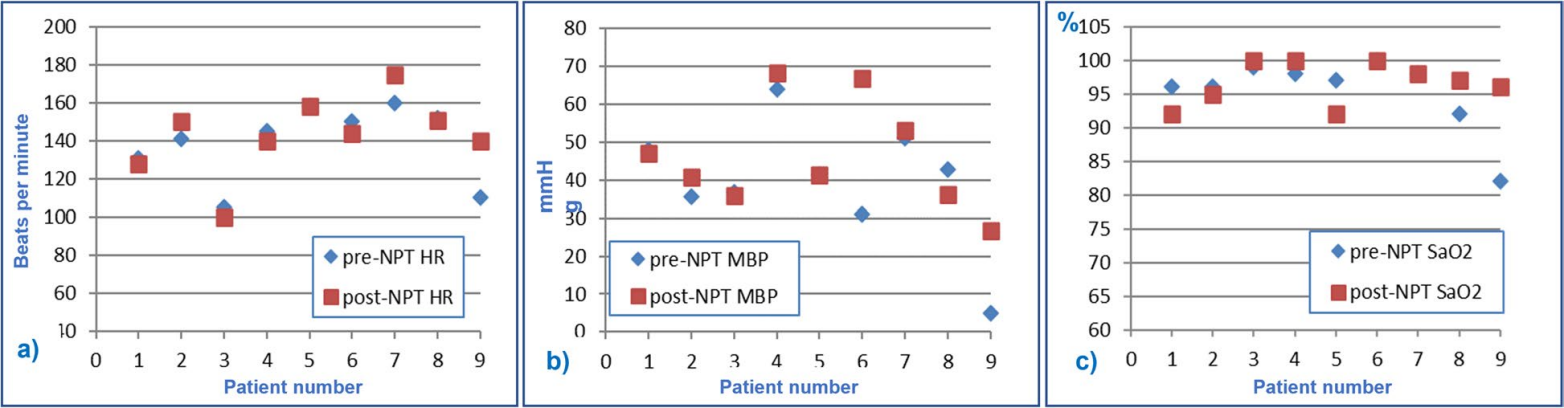
Graphic representation of vital parameters recorded before and after the transfer of infants treated with HFOV: **a** Heart Rate (HR) expressed in beats per minute, **b** Mean Blood Pressure (MBP) expressed in mmHg and **c** transcutaneous oxygen saturation rate (SaO_2_) in percentage (%)

**Fig. 3:**
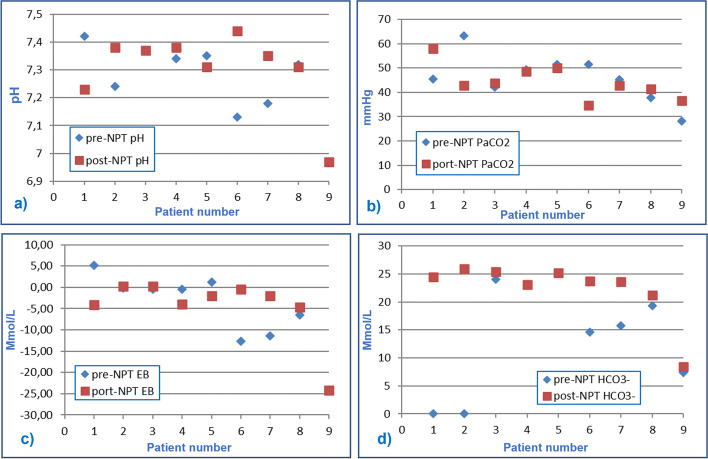
Graphic representation of hemogas-analytic values recorded before and after the transfer of infants treated with HFOV: **a** pH, **b** partial carbon dioxide pressure (PaCO_2_), expressed in mmHg; **c** excess bases (EB), in mmol/l; **d** bicarbonates (HCO3^−^), in mmol/l

**Fig. 4:**
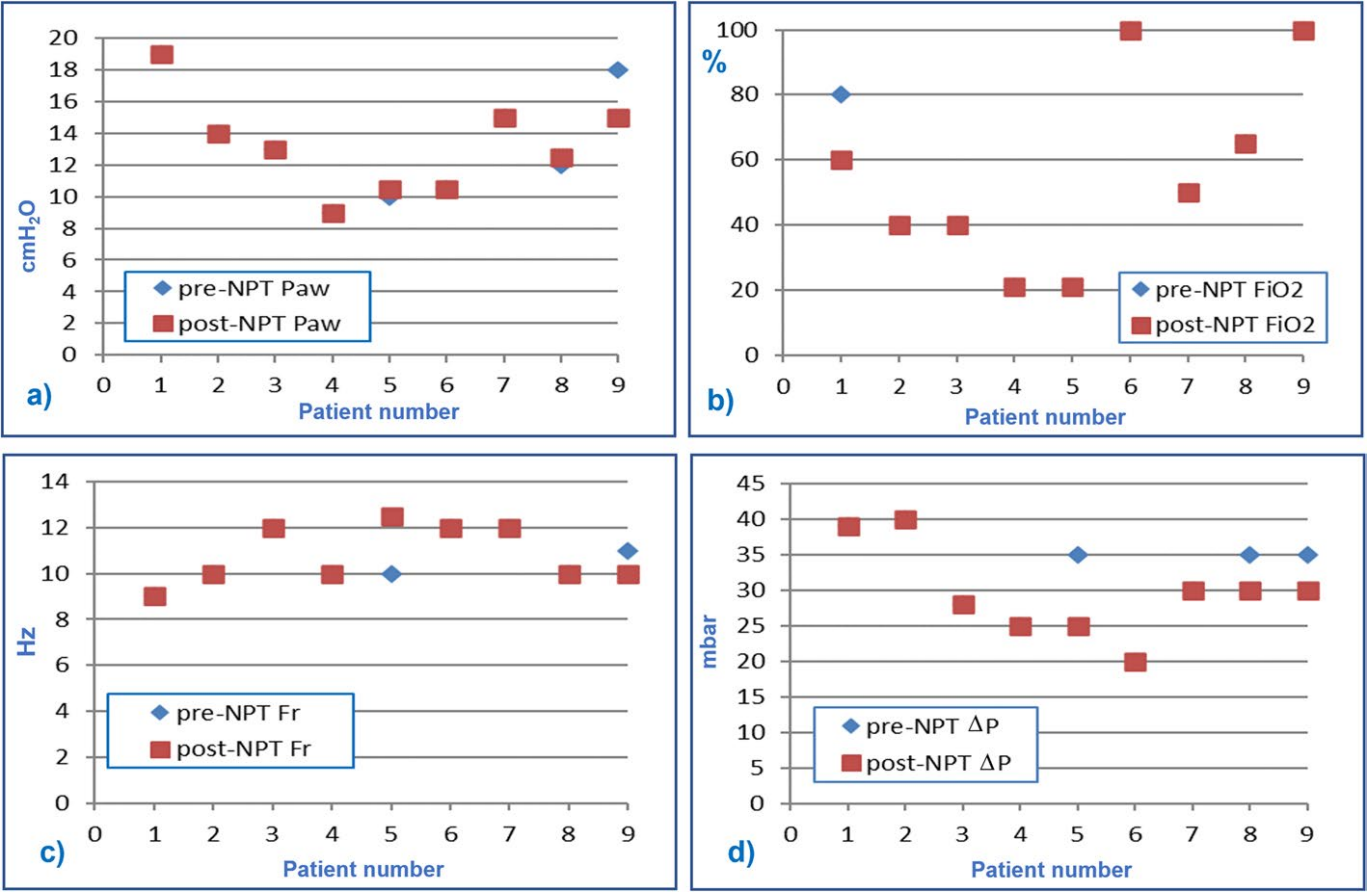
Graphic representation of changes in pulmonary ventilator settings recorded before and after the transfer of infants treated with HFOV: **a** mean airway pressure (Paw), expressed in cmH_2_0, **b** inspiratory fraction of oxygen (FiO_2_), expressed as a percentage; **c** oscillator frequency (Fr), in Hz; **d** Amplitude or Power (ΔP), in mbar

Table 2 shows the comparison between mean value ± standard deviation (DS) of vital parameters, hemogas-analytical values, and pulmonary ventilator settings of the nine patients before and after neonatal protected transport (pre-NPT and post-NPT). None of the parameters recorded showed significant differences between pre-NPT and post-NPT (*p* > 0,05). In addition, graphics have been drawn, to visually observe the trend of single variables for each patient (Figs. 2, 3 and 4).

**Table 2:** Comparison of Vital parameters, hemogas-analytical values and pulmonary ventilator setting recorded before and after neonatal protected transport

Vital Parameters	pre-NPT	post-NPT	*P*-value (paired t-test)
HR (bpm)	139 ± 20	143 ± 21	0,3767
MBP (mmHg)	39,5 ± 16,2	46,3 ± 14,2	0,1683
SatO_2_ (%)	95 ± 5	97 ± 3	0,4963
**Hemogas-analytic values**			
pH	7,26 ± 0,14	7,30 ± 0,14	0,7366
PaCO_2_ (mmHg)	46 ± 9	44 ± 7	0,6394
EB (mmol/l)	- 5,50 ± 9,20	- 4,54 ± 7,60	0,6727
HCO3- (mmol/l)	19,4 ± 6,33	22,3 ± 5,41	0,0749
**Setting of pulmonary Ventilator**			
P_aw_ (cm H_2_O)	13 ± 3	13 ± 3	0,5483
FiO_2_ (%)	57 ± 31	55 ± 29	0,3466
Fr (Hz)	10,6 ± 1,1	10,8 ± 1,3	0,6075
ΔP (mbar)	31,9 ± 6,6	29,7 ± 6,5	0,1037

Table 2: Vital parameters, hemogas-analytical values and pulmonary ventilator setting recorded before and after neonatal protected transport (pre-NPT and post-NPT), expressed as the average value ± standard deviation (DS)

The graphs in Fig. 2 show the values of vital signs detected before and after the transfer for each patient. HR remained in the normal range during HFOV transport in all patients. Overall, there was an increase, though not significant, in MBP recorded after the transfer. The blood pressure of patient 9 was not assessable at the time of departure, thus as pre-NPT value we conventionally used 5 mmHg. Upon arrival at NICU, the newborn's peripheral pulses became appreciable with MBP detected at 30 mmHg, thus suggesting a general clinical improvement. Only patient 8 showed a decrease in MBP, although it remained in the normal interval for gestational age. In all patients, SaO_2_% remained stable or increased during transport. In Patient 9, SaO_2_% increased by 14 percentage points, following the same pattern as MBP and HR, then confirming the overall hemodynamic improvement.

Figure 3 shows variations in hemogas-analytic parameters. Blood pH values remained broadly stable between pre-transfer and post-transfer detections: in 3 patients we noticed improvement in acid–base balance, while a decrease was relevant only in patient 1. In general, PaCO_2_ resulted persistently within the limits of normocapnia or permissive hypercapnia. Analyses of BE and bicarbonate variations yielded similar results, showing no significant variations pre-NPT and post-NPT. Five patients corrected the deficit or improved their condition. Patients 9, 4 and 5 showed minimal BE variations. The values of bicarbonates increased or remained unchanged in all patients except for patients 1 and 2 where they were not available because not performed at birthplace.

The setting of the ventilator remained unchanged during the transfer of patients in almost all cases, as shown in Fig. 4. Indeed, for patient 9 it was possible to reduce the Paw delivered. and in patient 1 it was possible to reduce FiO2 by 20% because of good Sa02 values during the transfer. The Fr remained mainly unchanged, except for minor variations in two patients. Accordingly, ΔP did not require modification in most cases, while a reduction was possible in three patients during transport.

Regarding the drugs used during transport, no patient needed additional inotropic medicaments or an increase in their dose, thus confirming the ongoing stability of hemodynamic status using HFOV during NPT. Four patients started Dopamine at birthplace and Dobutamine was infused during transfer to three patients only. Only two newborns received alkalizing therapy, because of the severity of acidosis; therapy started at birth point and no changes in dosage were necessary during the transfer. There has been no death or transport-related complication. All newborns reached our NICU treated with HFOV, and none needed to be converted to CMV. No re-entry transport was made to the transfer centre.

## Discussion

In this study we aim to assess the feasibility and safety of HFOV in the neonatal transfer of infants suffering from severe forms of respiratory failure. In the NETS of the central area of Tuscany this is delivered by the Oscillator Acutronic Fabian HFOi, part of a high-tech neonatal transport unit.

Indeed, when other modalities of respiratory support fail [12–14], the possibility of transporting a newborn in HFOV is beneficial, being an optimal rescue therapy, for its positive effects on gas exchange. In fact, HFOV is considered a lung protection strategy, because low tidal volume, constant mean airway pressure and high respiratory frequency provide beneficial effects on oxygenation and ventilation, reducing the risk of volotrauma and barotrauma [6]. Its benefits have been specifically reported in some respiratory diseases. For example, in the treatment of air-leak syndromes, such as pulmonary interstitial emphysema, pneumothorax, or pneumomediastinum, HFV is indicated as a form of gentle ventilation with low pressure and low tidal volume [8]. Various forms of HFV have been used to treat infants with lung air leak and the superiority of this type of ventilation over conventional ventilation has been demonstrated [25]. Keszler and collaborators showed that Jet Ventilation determined a faster improvement in ventilation in infants with Interstitial Pulmonary Emphysema, compared to CMV [26]. In 2017, Aurilia et al. used HFOV for conservative management of significant pneumothorax in preterm newborns hemodynamically stable and requiring mechanical ventilation [8]. This approach allowed reducing the deterioration of air leak and the insertion of chest tube drainage and all the subsequent associated risks [8]. HFOV has been shown to be effective also in treatment of persistent pulmonary hypertension of the newborn (PPHN) [15], especially when associated with iNO, achieving a significant increase in PaO_2_ and a fall in PCO_2_ [16]. Furthermore, HFOV is considered an important mean of respiratory support for infants with severe meconium aspiration syndrome (MAS) failing CMV [27]. Series published by large databases suggest that 20–30% of all infants with MAS requiring intubation and ventilation are treated with HFV [28, 29], especially HFOV. The latter appears particularly suitable in the alveolar recruitment of patients with severe atelectasis [30]. Hao and Wang in 2019 confirmed the effectiveness of HFOV in infants with MAS with reference to ventilator parameters at hemogas-analysis, as duration of hospital stay and absence of adverse events [7]. The effectiveness of HFOV has also been demonstrated in infants with severe MAS complicated with air leak [31] and, especially when associated with iNO, in infants with MAS developing PPHN [32, 33]. Regarding the use of HFOV in the treatment of congenital diaphragmatic hernia (CDH), the revised guidelines “Standardized Postnatal Management of Infants with Congenital Diaphragmatic Hernia in Europe” [17] recommends CMV as the optimal initial ventilation strategy for CDH treatment, but HFOV as a rescue therapy if CMV fails. In a study by Attar et al. in 2019 [18], HFOV used to rescue newborns with CDH, brought a significant improvement in ventilation and an adequate rate of decrease in PaCO2. In 2020 Gerall claimed that “High frequency oscillatory ventilation (HFOV) is a common rescue strategy for patients with congenital diaphragmatic hernia (CDH). Although shown to increase survival, HFOV, can also hinder care when transport is necessary” [34].

In literature, limited experience of the use of HFV in Neonatal Transport is available, mainly concerning other modes than oscillatory ventilation. In 2012, Zhang et al. published a retrospective study to validate the use of HFJV in neonatal transport of infants with prenatal diagnosis of CDH [20]. The authors noted a significant improvement in oxygenation and ventilation of patients undergoing HFJV and indicated the use of HFJV as the first choice for the safe transport of infants with CDH [20]. Another retrospective study, carried out by Mainali and colleagues, also demonstrated the efficacy and safety of HFJV associated with iNO, compared to CMV and iNO, in neonatal transport of infants with severe respiratory failure [22]. In their experience, HFJV could represent the first-choice mode of ventilation in these patients, for its ability to maximize the amount of CO2 eliminated. Honey et al. concluded on the efficacy and safety of HFPV in air transport using the “Duotron”, a pulmonary ventilator that allow HFPV [21]. Through the analysis of respiratory function and FiO_2_, the authors observed comparable values before and after transport and significant improvement in some patients [21].

To the best of our knowledge, the only study that investigated HFOV in neonatal transport is a Korean one, published by Lee in 2012, where the Sophie STEPHAN® lung ventilator was adopted, able to provide HFOV and characterized by an internal 24 V DC battery [23]. Lee used HFOV during transport in two occasions. In one case, however, the ambulance's electrical supply failed to meet the pulmonary ventilator's demands, forcing operators to abandon the oscillator and convert the patient to manual ventilation. Hence, the study quantified the autonomy of the HFOV transport device, detecting an average battery life of about 60 min [23]. The transport system with Sophie HFO was therefore approved for transfers of less than one hour of travel [23]. Another experience, although regarding infants with a median age of 26 days (interquartile range 8–151 days), derived from a retrospective study conducted by Chassery in 2015 [35]. The report compared the use of CMV with HFOV, supplied with the Leoni Plus ventilator, in the inter-hospital transport of patients with severe bronchiolitis HFOV, concluding that HFOV is a useful and effective tool when transporting these patients [35].

The aim of our work has been to overcome the technical difficulties shown in previous experiences. The patients in our study, characterized by considerable variability in terms of gestational age and birth weight, suffered from severe respiratory failure because of medical or surgical pathology, which imposed the transfer from birth hospital to our NICU. The relatively short mean transport time (about 20 min with a maximum of 45 min), which could be considered a limit of our study, was due to the short distance between the different hospitals, not to restrictions in autonomy of the ventilator when used in HFOV. Indeed, the need to operate independently is limited to the transfer phase from the requesting hospital to the ambulance, while during the journey in ambulance the supply of the oscillator depends on the current generator and the medical gas sources of the vehicle.

In our experience, no statistically significant changes of vital parameters (HR, SaO2 and MBP) were detected before and after the transfer. Moreover, these parameters showed a positive trend in a newborn, who suffered from severe hypovolemic shock due to untimely placental abruption, thus suggesting an overall improvement in clinical condition. Among the hemogas-analytical parameters, the pH of patients remained substantially stable, showing an improvement of acidosis in three infants. pH of two newborns, on the other hand, showed a negative trend. Nevertheless, in only one case the decrease in pH was significant and concomitant with a slight increase in PaCO_2_ and reduced bases. However, this patient was an overly complex case, suffering from a malformative syndrome in addition to severe prematurity. On the contrary, PaCO_2_ decreased in two patients during the journey and in other cases, the values remained constant and within the limits of permissive hypercapnia. Overall, no changes in ventilator settings were necessary during the transfer, except in some cases when we could reduce the respiratory support. In fact, it was possible to reduce the FiO_2_ in one case and ΔP in three patients, due to improved gas exchanges. In all cases, there were no inotropic drugs administered in addition to those given at birthplace. The NETS team was never forced to stop during the trip and return to the hospital of reference, due to hemodynamic stability or improvement of the patients. Conversion from HFOV to CMV was never necessary and all infants were admitted to NICU in HFOV. There was no obstruction or dislocation of the endotracheal tube, no new-onset air leaks and no loss of visible vibrations on the chest. Therefore, altogether, these data confirm the safety of the use of HFOV in neonatal transport.

Our study is limited to a small number of infants, but cases of severe and refractory respiratory failure, which can benefit from HFOV in NETS, are fortunately rare and the system has been in use only since May 2018. Some data is also incomplete, due to the retrospective nature of the study and the difficulties of an emergency setting such as neonatal transport.

## Conclusions

In conclusion, the interest of our report is in the possibility of using HFOV during inter-hospital neonatal transfer, since we have realized a novel neonatal transport unit capable of providing HFOV without time and autonomy limit, thus overcoming the previously existing technical difficulties. In our experience, HFOV has proven to be an effective and safe instrument for transport of newborns with severe respiratory failure, either already assisted with this type of ventilation at the hospital of birth or, in some cases, stabilized by the NETS team in HFOV, because little or no response to CMV. The opportunity to continue HFOV during transport is a great advantage for these newborns, avoiding unnecessary shift from one kind of ventilation to another and thus preventing hemodynamic and respiratory instability. Nevertheless, due to the low number of patients, further larger-scale, prospective and multicentre studies, will be necessary to confirm the safety and efficacy of HFOV during neonatal transport.

## Data Availability

All data generated or analysed during this study are included in this published article.

## Abbreviations

HFOV: High Frequency Oscillatory Ventilation
NICU: Neonatal Intensive Care Unit
NETS: Neonatal Emergency Transport Service
CMV: Conventional Mechanical Ventilation
PPHN: Persistent pulmonary hypertension of the newborn
MAS: Meconium aspiration syndrome
CDH: Congenital diaphragmatic hernia
HFV: High Frequency Ventilation
HFJV: High Frequency Jet Ventilation
HFPV: High Frequency Percussive Ventilation
iNO: Inhaled nitrogen monoxide
NPT: Neonatal Protected Transport
HR: Heart rate
MBP: Mean blood pressure
SaO2: Oxygen saturation rate of haemoglobin
PaCO2: Partial carbon dioxide pressure
EB: Excess bases
Paw: Mean airway pressure
FiO2: Inspiratory fraction of oxygen
Fr: Frequency of oscillations
ΔP: Amplitude or Power of oscillations
DS: Standard deviation
